# Surgical treatment of cranial cruciate ligament disease in dogs using Tibial Plateau Leveling Osteotomy or Tibial Tuberosity Advancement–A systematic review with a meta-analytic approach

**DOI:** 10.3389/fvets.2022.1004637

**Published:** 2022-11-30

**Authors:** Annika Christina Wemmers, Marios Charalambous, Oliver Harms, Holger Andreas Volk

**Affiliations:** Department of Small Animal Medicine and Surgery, University of Veterinary Medicine Hannover, Hannover, Germany

**Keywords:** canine (dog), orthopedic disease, stifle surgery, cranial cruciate ligament rupture, Tibial Plateau Leveling Osteotomy (TPLO), Tibial Tuberosity Advancement (TTA)

## Abstract

Tibial Plateau Leveling Osteotomy (TPLO) or Tibial Tuberosity Advancement (TTA) are commonly used surgical techniques for correction of cranial cruciate ligament (CCL) rupture in dogs. This systematic review aims to investigate whether one technique is superior to the other. Seventy-two studies on surgical management of CCL rupture have been identified and evaluated in regard of subjective and objective gait analysis criteria, development of osteoarthritis (OA), thigh circumference measurements, goniometry, joint stability, pain and complication rates. Almost half (47.2 %) of the studies were considered of low quality of evidence, leading to high heterogeneity in quality among studies; this posed a major limitation for an evidence-based systematic review of both surgical techniques. Out of 72 studies, there were only eleven blinded randomized clinical trials, of which five were rated with a low overall risk of bias. However, both techniques were considered to be successful management options. Subjective and objective gait analysis revealed no lameness at long-term evaluation for the majority of the patients. However, it appeared that TTA lead to better OA scores up to 6 months postoperatively, while TPLO had a lower rate of surgical site infections. In summary, no method can be clearly preferred, as most of the study evaluated were subpar. Studies with a high level of evidence are therefore urgently needed for such a common surgical procedure.

## Introduction

Cranial cruciate ligament (CCL) rupture is one of the most frequent causes for pelvic limb lameness in dogs (1) posing substantial economical and clinical consequences (2). The origin and development of CCL disease has been widely discussed in many studies (3–8). Currently, a variety of surgical treatment options have been developed, with Tibial Plateau Leveling Osteotomy (TPLO) and Tibial Tuberosity Advancement (TTA) being well established and frequently used surgical procedures (9, 10). Both techniques produce a biomechanical alteration and, thus, stabilization of the stifle joint (11, 12).

The clinical outcomes of TPLO and TTA have been studied and compared with respect to various clinical parameters (13). A previous systematic review (13) suggested that TPLO achieves better results in objective gait analyses in restoring limb function, while subjective gait analyses found no differences between TPLO and TTA. At first glance, the analysis of the multiple studies allows the hypothesis that TPLO is superior to TTA in terms of long-term clinical outcomes, although further research and systematic analyses of the data to date are needed to confirm this.

The outcome of stifle joint osteotomies can be assessed clinically and radiographically, while the occurrence or rate of complications can be used as an additional outcome measure. Clinically, the gait can be assessed with both subjective and objective criteria. The subjective evaluation is based on a scale ranging between severe (no-weight-bearing) to normal gait (14, 15). Objective gait evaluation is based on the force gait plate analysis (16). This approach is less prone to bias and allows objective assessment of even non-visible changes in gait (17). Functional outcome also includes thigh circumference measurements, goniometry, joint stability and pain as these parameters provide information on the joint's mobility (18–21).

Surgical intervention leads to radiographically detectable osteoarthritis (22–26). Osteotomies are highly invasive surgical procedures and carry the risk of multiple complications associated with functional limitations and the need of further therapy (27, 28). Hence, it is necessary to assess the complication rates and most common causes of complications with the aim to address them in the future.

This systematic review aims to compare TPLO and TTA with regard to various outcome criteria and to evaluate the most current studies (2016–2021) on both surgical techniques. Specifically, we hypothesize that:

- TPLO has a better functional outcome on subjective and objective gait analysis than TTA
- TPLO has a better radiographical outcome on osteoarthritis than TTA
- TPLO has a better functional outcome on thigh circumference measurements than TTA
- TPLO has a better functional outcome on goniometry than TTA
- TPLO has a better functional outcome on joint stability than TTA
- TPLO has a better functional outcome on pain than TTA
- TPLO results in less complications than TTA.

## Materials and methods

### Search strategy

Several electronic databases have been searched to identify studies examining clinical outcomes of TPLO and/or TTA surgery. Search was performed from May 2021 until July 2021 using the terms “[cranial cruciate ligament AND (dog OR canine)] OR [cranial cruciate ligament AND (dog OR canine) AND surgery] OR [cranial cruciate ligament AND (dog OR canine) AND complication] OR [cranial cruciate ligament AND (dog OR canine) AND injury] OR [stifle AND surgery AND (dog OR canine)] OR (osteotomy AND stifle AND dog) OR” Tibial Plateau Leveling Osteotomy “OR” Tibial Tuberosity Advancement “OR Kreuzband Hund OR Kreuzbandriss Hund OR TPLO OR TTA.” Electronic databases included Pubmed, Web of Science, CAB Abstracts and the catalog of the library of the University of Veterinary Medicine Hannover.

A two-stage screening process was performed to identify studies for inclusion (29). Firstly, the search identified studies of relevance that seemed to fulfill inclusion criteria after screening titles and abstracts. Also, articles referenced in studies have been elected for further analysis. After searching databases, the full texts of the selected studies were examined in detail and included or excluded depending on the following criteria (stage 1):

#### Inclusion and exclusion criteria

- Type of study: Peer reviewed studies written in English or German, full text available, published between February 2016 and July 2021 were included. Clinical studies, case series, cohort studies, case reports, case-control studies and observational studies were included. *Ex-vivo* or experimental studies, literature reviews and studies with <5 subjects per group were excluded.
- Case definition: Patients diagnosed with CCL disease and eligible for surgery were included. Studies that investigated CCL disease plus any comorbidity were excluded whenever the second disease has been an obligatory inclusion criterion (e.g., dogs with amputated limbs plus CCL disease in another limb).
- Intervention: Only studies describing TTA or TPLO were included. Studies were excluded if dogs received another surgical procedure (e.g., combined TPLO and tibial tuberosity transposition).
- Outcome: Studies examining results on subjective lameness evaluation, objective lameness evaluation, osteoarthritis, thigh circumference measurements/muscle atrophy, goniometry, joint stability, pain and complications were included. Studies that did not differentiate between the results of each outcome parameter (e.g., using one score for several outcomes and not stating each outcome) were excluded.

Stage 2 consisted of a detailed evaluation of the studies' full texts regarding the studies' quality of evidence and treatment outcomes. Treatment outcomes have been evaluated as described in “Assessment of outcome measures.”

### Assessment of quality of evidence

Several factors have been identified to contribute to the overall quality of evidence of each study. Assessment of quality of evidence has been performed for all included studies. Quality assessment is based on the study design (blinding, randomization, use of a control group), study group size, duration of the study, subject enrolment quality/disease characterization and the assessment of risk of bias.

#### Study design

Studies were categorized into three groups providing different quality of evidence. The first group (Group A) included blinded randomized clinical trials (bRCTs) and non-blinded randomized clinical trials (nbRCTs) and was considered to produce high quality of evidence (the bRCTS were considered as the studies with the highest quality of evidence). To meet conditions for Group A, studies have been examined on their level of blinding, methods of randomization and use of a control group. Clinical trials not fulfilling these conditions were categorized in Group B, i.e., non-randomized clinical trials (NRCTs) and uncontrolled clinical trials (UCTs). These studies were considered to produce less quality of evidence than bRCTs. The third group (Group C) includes case series (CS), case reports (CR), cohort studies (CoS), case-control studies (CCS), observational studies (ObS) and all other types of non-interventional studies. They are considered to produce the lowest quality of evidence.

#### Study group size

Based on the study group sizes, studies were defined as “good” when >50 subjects per group, “moderate” when 20–50 subjects per group, “small” when 10–19 subjects per group and “very small” when <10 subjects per group were included (29). Studies with <5 subjects per group were excluded. Moreover, subject in terms of CCL surgery is defined as dogs enrolled in the study, not by knees that TPLO or TTA has been performed on. Bilateral surgery counts as one subject.

#### Duration of the study

Outcome parameters were evaluated at different time frames following surgery. Study duration is defined as the time between surgery (t0) and last examination (t1). Studies were categorized as “short-term,” i.e., up to 8 weeks, “mid-term,” i.e., 8 weeks−6 months and “long-term,” i.e., >6 months.

#### Subject enrolment quality/disease characterization

Disease characterization was performed by confirming the rupture of the CCL. The subjects' enrolment quality was considered to be “well,” “fairly,” “poorly” or “unclear” based on the following examination:

- “well” = patients underwent clinical and orthopedic examination (lameness evaluation, cranial drawer test, tibial thrust) AND x-ray of the affected joint AND confirmation of the rupture *via* MRI or arthroscopy or arthrotomy
- “fairly” = patients underwent clinical and orthopedic examination (lameness evaluation, cranial drawer test, tibial thrust) AND x-ray of the affected joint
- “poorly” = patients underwent clinical and orthopedic examination (lameness evaluation, cranial drawer test, tibial thrust)
- “unclear” = no or indistinct information given on the confirmation of CCL rupture.

#### Assessment of risk of bias

The risk of bias was addressed using the Cochrane Handbook for Systematic Reviews of Interventions tool (30). Potential sources of bias were selection bias (sequence generation, allocation concealment), performance bias (blinding of participants and personnel), detection bias (blinding of outcome assessment), attrition bias (incomplete outcome data), reporting bias (selective reporting) and other bias such as conflicts of interests. Each element was characterized as providing “high,” “moderate,” “unclear” or “low” risk of bias (scale: 3 = high risk of bias, 2 = moderate or unclear risk of bias, 1 = low risk of bias). These numbers were summed up to a total score and, hence, the studies were further categorized on the grounds of their overall risk of bias score:

- Score 7–9 = overall “low” risk of bias
- Score 10–12 = overall “low/moderate” risk of bias
- Score 13–15 = overall “moderate” risk of bias
- Score 16–18 = overall “moderate/high” risk of bias
- Score 19–21 = overall “high” risk of bias.

### Assessment of outcome measures

The outcome of TPLO or TTA surgery can be measured using the following parameters: gait analysis (subjective), gait analysis (objective), osteoarthritis, thigh circumference measurements, goniometry, joint stability, pain and complications. At least one of these parameters should have been assessed in each included study. The efficacy of TPLO and TTA was evaluated based on the aforementioned outcome criteria and the timing of the last examination (short-term, mid-term and long-term results; pre-operative vs. post-operative). One standardized scale was applied to each outcome criterion so that the results can be compared and evaluated in a homogenous process.

#### Gait analysis (subjective)

The following scale was used to assess the gait analysis in each study:

- 0 = no lameness
- 1 = mild lameness
- 2 = moderate lameness
- 3 = severe lameness
- 4 = non-weight bearing lameness.

The outcome was also characterized based on the time frame, i.e., short-term (up to 8 weeks) and mid-term (up to 6 months):

- No lameness (score </= 0.5)
- Improvement (in %) of a minimum of 50 % (as decrease in lameness score between preoperative and postoperative evaluation).

#### Gait analysis (objective)

The objective gait analysis was performed (i) either on a force plate or pressure platform and ground reaction forces were expressed as peak vertical force and vertical impulse or (ii) on a static stance analyser and were expressed in percent of body weight on all four legs. The outcome was also characterized based on the time frame, i.e., short-term (up to 8 weeks) and mid-term (up to 6 months):

- Significant improvement between preoperative and postoperative evaluation
- Improvement (in %) of a minimum of 30 % between preoperative and postoperative evaluation to account for a potential placebo effect and to ensure clinical efficacy.

#### Osteoarthritis

Osteoarthritis was evaluated based on radiographic diagnostic images by a veterinary clinician. The following scale was used:

- 0 = no OA
- 1 = mild OA
- 2 = moderate OA
- 3 = severe OA.

The outcome was also characterized based on the time frame, i.e., short-term (up to 8 weeks) and mid-term (up to 6 months):

- No OA (score </= 0.5) at mid-term or long-term evaluation
- Progression (in %) of a maximum of 10 % (>/=) between preoperative evaluation and mid-term or long-term evaluation.

#### Thigh circumference measurements

The thigh circumference was measured with a tape measure by a veterinary clinician. The reported outcome of each study was expressed either as symmetry index (ratio in thigh circumference between the affected and the healthy hind limb) or as the difference in thigh circumference in the affected limb pre-operatively and post-operatively.

Outcome was considered successful with a post-operative symmetry index > 0.95 or when there was no significant loss in muscle mass between pre-operative and post-operative measurements.

#### Goniometry

ROM (Range of Motion) or flexion and extension in degree was measured with a goniometer by a veterinary clinician or fluoroscopic images. Outcome was considered successful when the ROM or flexion or extension was increased pre-operative compared to post-operative level or when there was no difference between the healthy and affected pelvic limb at the final post-operative examination.

#### Joint stability

Joint stability was assessed either by a fluoroscopic examination or the tibial compression test. The outcome was considered successful when the limb was stable, i.e., negative in tibial compression test or no abnormalities, such as femorotibial craniocaudal translation, shown in fluoroscopy.

#### Pain

The pain score was assessed by a veterinary clinician and expressed using a validated instrument (standardized questionnaire), or using a scale or simply stating “pain is present” or “pain is absent.” The following scale was used:

- 0 = no pain
- 1 = mild pain
- 2 = moderate pain
- 3 = severe pain.

The outcome was also characterized based on the time frame, i.e., short-term (up to 8 weeks) and mid-term (up to 6 months) and considered successful when pain score </= 0.5.

#### Complications

Operation-related complications were categorized into major (any complication that needed further surgical intervention) or minor (any complication that did not require surgical intervention). A distinction in complication rates based on the size (mean body weight) of the dogs in each study was also used:

- Small dogs (group S): < 15 kg
- Medium dogs (group M): 15–40 kg
- Large dogs (group L): > 40 kg.

### Statistical analysis (meta-analytic approach)

A Mann-Whitney-U-Test was calculated to determine if there were differences in mean osteoarthritis scores between TPLO and TTA. For the comparison groups' studies, a further approach was conducted to identify statistical differences between studies with regards to reported complications. For each study, the total number of surgeries with reported complications in the treated groups (TPLO vs. TTA) were retrieved. The odds ratio (OR) was then estimated in order to indicate the increased or decreased odds of observing a specific complication for the TPLO compared to TTA. Statistical analysis was undertaken following the guidelines of the Handbook of the Cochrane Collaboration 5.4.1. The OR for dichotomous data was calculated using the random-effects model in Review Manager 5.4.1. Heterogeneity between studies was calculated using the Chi square test and was considered to be heterogeneous when *P* ≤ 0.1. I2 values of <25, 26–74, and >75 % were considered as “low,” “moderate” and “high” heterogeneity, respectively. Associations were considered to be statistically significant at *P* < 0.05.

## Results

### Description of studies

The search identified a total number of 72 studies fulfilling the inclusion criteria (see Supplementary material). The database searches delivered about 7,000 results and after screening titles, abstracts and full texts for keywords, the number of eligible studies was 156. Then, 84 of these studies were excluded due to a lack of detailed results, investigations on CCL disease plus comorbidities or indistinct definition of which orthopedic surgery has been performed, which left a total of 72 studies for inclusion. Two studies have been published twice (31–34) with the same study population and study design, but each paper focussed on different outcome parameters.

### Overall quality of evidence

#### Study design

Less than half (34 studies, 47.2 %) of the studies was assigned to group C and considered to have least overall quality. There were 20 studies (27.8 %) in group B and 18 studies (25.0%) in group A. Out of 18 studies in group A, there were eleven bRCTs, of which five were rated with a low overall risk of bias (35–40) (see Supplementary material).

#### Study group size

Information on study group size was provided in all of the included studies. However, the definition of “cases” or “subjects” was inconsistent between studies. The authors counted the number of surgeries per subject for evaluation, hence, dogs with a bilateral CCL rupture were counted as two independent cases or subjects. Others differentiate between the number of dogs they included and the number of surgeries performed. Some authors (31–34, 36–38, 41–57) only included dogs with unilateral CCL rupture, so the number of dogs and number of cases were the same. Study group sizes varied between five subjects per group (56, 58) and 1,732 subjects per group. The highest total number of subjects in one study was 1,732 dogs with 1,768 surgeries (59).

#### Duration of the study

Study duration varied from 3 days (37) up to 5 years (58, 60–64). Twenty-eight, 26 and 18 studies evaluated mid-term, short-term and long-term outcomes, respectively.

#### Subject enrolment quality/disease characterization

The majority of the studies (36 studies) provided detailed information based on common and validated methods for the diagnosis of CCL disease. Their subject enrolment quality was therefore considered of high standard. Eight studies diagnosed CCL disease “fairly” and one study did poorly. There were 27 studies that provided unclear information on how diagnosis of CCL disease was confirmed.

#### Assessment of risk of bias

##### Randomization

All studies in group A used randomization to allocate subjects to groups. Random sequence generation and allocation concealment were evaluated to assess selection bias. Twelve studies were considered to produce low risk of selection bias (Table 1) regarding random sequence generation and allocation concealment (as they used adequate methods to generate the allocation sequence: these studies assign subjects *via* computer-assisted lists (31, 36, 37, 39, 48, 54, 65, 66) such as Excel or commonly available online tools, *via* coin toss (35, 38, 44) or by referring to a random number table (40).

**Table 1:** Risk of bias.

**Random sequence generation (selection bias)**	**Allocation concealment (selection bias)**	**Blinding of participants and personnel (performance bias)**	**Blinding of outcome assessment (detection bias)**	**Incomplete outcome data addressed (attrition bias)**	**Selective reporting (reporting bias)**	**Other bias**
Low	Low	Low	Low	Low	Low	Low
12	13	7	11	20	19	11
16.67 %	18.06 %	9.72 %	15.28 %	27.78 %	26.39 %	15.28 %
Unclear	Unclear	Unclear	Unclear	Unclear	Unclear	Unclear
8	5	4	10	37	37	23
11.11 %	6.94 %	5.56 %	13.89 %	51.39 %	51.39 %	31.94 %
High	High	High	High	High	High	High
52	54	61	51	15	16	38
72.22 %	75.00 %	84.72 %	70.83 %	20.83 %	22.22 %	52.78 %

Risk of bias assessment presented as absolute numbers and percentages across all included studies.

Red, High risk of bias; Yellow, Unclear risk of bias; Green, Low risk of bias.

Three studies only stated that they “randomly assigned” (32, 49) or used a “simple randomization procedure” (51) without giving detailed information on the procedure applied. One study (47) assigned subjects *via* a randomized block cohort study on the basis of patient-related data that might had introduced a source of bias.

One study (67) assigned dogs to a treatment group alternately and another study (68) used pre-labeled notecards in a pile. Both did not give information on how allocation concealment was guaranteed.

A very uncommon methodology of comparing two surgical treatments was used in one experimental study (69) that artificially transected the cranial cruciate ligament. Specifically, each hindlimb was randomly assigned (*via* coin toss) to receive either a TTA or TPLO technique. Every dog was treated with TTA on one leg and TPLO on the contralateral. The comparison between TTA and TPLO was based on the evaluation of limbs of the same dog, rather than between different dogs. This study was considered a NRCT.

##### Blinding

Eleven studies were categorized as blinded, however only seven (35, 36, 38–40, 47, 54) of them described in detail a complete blinding of patients, personnel and outcome assessors. They were therefore considered to have a low risk of performance and detection bias (Table 1). Some studies described blinding to some extent (37, 65) or it was not possible to blind all personnel and owners to all treatments (31, 32).

##### Incomplete outcome data

Low risk of attrition bias was attributed to 27.8 % of the studies as they reported either no missing outcome data or no reasons for missing outcome data which could affect the outcomes (Table 1). In 51.4 % of the studies, insufficient information was provided on whether all included dogs completed the trial until the end or it was unclear whether missing outcome data affected the outcomes. 20.8 % of the studies had a high risk of attrition bias. In retrospective studies, in particular, it was a common limitation for the study that dogs were not even included since no follow-up examination was possible; the reasons for this could be related to the outcome of the treatment, i.e., when it is unsatisfactory and the owners might opt for a re-evaluation in another veterinary clinic.

##### Selective reporting

Most studies (51.4 %) deliver an unclear risk of bias as they did not correctly report every detail concerning study design and outcome data. Unclear or high risk of bias was also seen in studies (54, 56, 60–64, 68, 70–77) that were (partly) based on results stated by the owners or referring vets (Table 1). These might not have followed standard protocols, but be influenced by the owners' or referring vets' personal opinion and bias toward the treatment.

##### Other bias

In several studies, other potential sources of bias have been identified (Table 1). Many studies were of retrospective nature (42, 50, 52, 53, 55, 56, 58–61, 63, 64, 71–95) which imposes a high risk of bias as protocols have not been standardized beforehand. Also, reported outcomes which might be different from the true outcome as potential follow-up losses cannot be identified. Studies with unclear financial support (62, 63, 83, 87, 96, 97), support of financial nature, consultant activity, company collaboration or speaker engagement (31–33, 36–38, 40, 44, 46, 47, 56, 60, 65, 69, 71, 72, 76, 98) (e.g., financial funding) or other potential conflicts of interests (39, 50, 51, 54, 57, 59, 67, 73, 80, 92) led to an unclear risk of bias.

### Intervention

A total of 72 studies were included in this review. Forty-six studies examined the results of TPLO surgery, 20 studies focused on TTA surgery and six studies considered TPLO and TTA outcome. In addition to the surgical intervention itself, 24 (20 TPLO, four TTA) studies evaluated the results of additional (postoperative) treatments or other factors that contributed to surgical outcome.

### Outcome

Number of studies for each outcome parameter can be found in Table 2.

**Table 2:** Outcome parameters.

	**Gait analysis (subjective)**	**Gait analysis (objective)**	**Osteo-arthritis**	**Thigh circum-ference**	**Goniometry**	**Joint stability**	**Pain**	**Complications**
TPLO	11	13	7	5	4	4	6	36
TTA	9	1	4	3	3	1	5	17
TPLO + TTA	2	2	3	0	0	1	0	5
Total	22	16	14	8	7	6	11	58

Number of studies that investigated on the outcome parameters subjective gait analysis, objective gait analysis, osteoarthritis, thigh circumference, goniometry, joint stability, pain, and complications.

#### Gait analysis (subjective)

Twenty-two studies with a total of 876 surgeries (349 TPLO, 527 TTA) evaluated the outcome of CCL surgery based on this criterion. Eleven studies reported TPLO outcome, nine studies TTA outcome and two studies compared results of TPLO und TTA surgery. The quality of evidence varied between TPLO and TTA studies. High quality of evidence was provided in eight studies on TPLO and only one study on TTA.

At short-term evaluation, four of eleven (36 %) TPLO treatment groups (36, 38, 51, 78) and two of seven (29 %) TTA treatment groups (70, 99) showed a successful outcome regarding a lameness score </= 0.5 (no lameness) (Table 3). The mean lameness score for TPLO was 0.87 (median 0.71) and 0.83 (median 0.7) for TTA. Lameness scores ranged from 0.3 to 2.4 for TPLO and 0.3–1.6 for TTA. Four of nine (44 %) TPLO treatment groups (36, 38, 51) and four of six (67 %) TTA treatment groups (55, 70, 99) had successful outcome with an improvement of a minimum of 50 % between pre-operative and short-term postoperative evaluation (Table 3). Improvement varied between 0–72.5 % for TPLO and 20–81.3 % for TTA.

**Table 3:** Subjective lameness at short-term evaluation.

**Short-term** ** evaluation**	**Procedure**	**No. operated joints**	**Eligible study groups w/ available data (number of high quality studies)**	**Study groups w/ successful outcome (number of high quality studies)**	**Study groups w/ successful outcome in % (number of high quality studies)**
Lameness Score	TPLO	207	11 (8)	4 (3)	36 (38)
	TTA	246	7 (1)	2 (0)	29 (0)
Improvement	TPLO	163	9 (8)	4 (4)	44 (50)
	TTA	233	6 (1)	4 (0)	67 (0)

Total number of operated joints, eligible study groups and study groups with successful outcome for each procedure for the criteria “Lameness score” and “Improvement,” as well as percentage of study groups with successful outcome out of all eligibly study groups. Results when considering only study groups from high quality (A) studies are listed in brackets.

The mid-term outcome was successful in five out of six (83 %) TPLO treatment groups (48, 51, 67, 69) and five out of five (100 %) TTA treatment groups (79, 99, 100) showing no lameness. Five out of five (100 %) TPLO treatment groups (48, 51, 67) and three out of four (75 %) TTA treatment groups (99, 100) had successful outcome with an improvement of a minimum of 50 % between pre-operative and mid-term postoperative evaluation (Table 4). The mean lameness score for TPLO was 0.16 (median 0.09) and 0.09 (median 0.12) for TTA. Lameness score ranged between 0–0.6 for TPLO and 0–0.2 for TTA and improvement between 50–61.5 % and 49.1–85.5 % for TPLO and TTA, respectively.

**Table 4:** Subjective lameness at mid-term evaluation.

**Mid-term evaluation**	**Procedure**	**No. operated joints**	**Eligible study groups w/ available data (number of high quality studies)**	**Study groups w/ successful outcome (number of high quality studies)**	**Study groups w/ successful outcome in % (number of high quality studies)**
Lameness Score	TPLO	98	6 (4)	5 (3)	83 (75)
	TTA	280	5 (1)	5 (1)	100 (100)
Improvement	TPLO	83	5 (4)	5 (4)	100 (100)
	TTA	265	4 (1)	3 (1)	75 (100)

Total number of operated joints, eligible study groups and study groups with successful outcome for each procedure for the criteria “Lameness score” and “Improvement,” as well as percentage of study groups with successful outcome out of all eligibly study groups. Results when considering only study groups from high quality (A) studies are listed in brackets.

When only high-quality studies were compared, only one could be assessed for TTA showing a better mid-term result, but no information was available for short-term success, whereas the short- and mid-term results were similar for TPLO (Tables 3, 4).

Overall, the studies provided evidence that both TPLO and TTA surgery were highly effective in treating CCL disease regarding the subjectively evaluated lameness. For both procedures, lameness scores decreased continuously and resulted in “no lameness” 6 months postoperatively in all treatment groups, except in one TPLO surgery group.

#### Gait analysis (objective)

Objective gait analysis was performed in 16 studies: 13 studies evaluated the outcome of TPLO, one of TTA and two studies compared TPLO and TTA outcome. Overall, 519 surgeries (453 TPLO, 66 TTA) on CCL disease with concurrent objective gait analysis were reported. High quality of evidence was provided in nine studies on TPLO and in one study on TTA.

Regarding the short-term assessment: For TPLO, several studies on objective gait parameters already showed a significant improvement (31, 46, 48, 66, 67) [16 of 22 (73 %) study groups with successful outcome] as well as a clinically visible improvement of at least 30 % (38, 40–42, 47) [17 of 38 (45 %) study groups with successful outcome]. For TTA surgery, there was only one eligible study providing sufficient information on significant differences pre- and postoperatively. This study is of high quality of evidence and reports a significant improvement (48). The two studies reporting on the overall improvement showed that there was no improvement of more than 30 % in the different gait parameters (45, 48).

At mid-term evaluation, all studies reported a significant improvement in ground reaction forces in all (100 %) groups compared to pre-operative assessment and therefore a successful outcome on TPLO as well as TTA surgery (31, 40, 42, 45, 48, 60, 67). This result was consistent with subjective gait analysis.

Overall, the studies provided evidence that both TPLO and TTA surgery were highly effective in treating CCL disease regarding the objectively evaluated lameness up to 6 months after surgery. However, both studies with high and low quality of evidence were combined for attaining this comparison.

#### Osteoarthritis

Fourteen studies investigated the development of osteoarthritis after CCL surgery with seven studies evaluating TPLO surgery, four studies evaluating TTA surgery and three studies comparing the outcome between TPLO and TTA. A total of 447 surgeries (329 TPLO, 148 TTA) have been performed. High quality of evidence was provided in six studies on TPLO and in one study on TTA.

The majority of the studies (32, 36, 48, 51, 61, 62, 67, 69, 84, 91) focused on short- and mid-term outcome, whereas only three authors (49) considered long-term outcomes.

All studies with available outcome data reported the occurrence of osteoarthritis postoperatively as relatively severe. At short- and mid-term evaluation, four out of twelve (33 %) TPLO study groups (32, 36, 61) and three out of seven (43 %) TTA study groups (62, 91) showed a progression in osteoarthritis of <10 % and were therefore considered successful. Mild osteoarthritis was seen in seven (58 %) TPLO (32, 48, 61, 67, 69) and seven (100 %) TTA study groups (48, 61, 62, 69, 84, 91), moderate osteoarthritis also in five (42 %) TPLO study groups (32, 36, 51). The mean OA score was 1.44 (median 1.5) for TPLO and 0.96 (median 0.9) for TTA.

Long-term outcome was not successful in any of the evaluated studies (49, 61, 62), since all the osteoarthritis scores showed at least mild osteoarthritis and a progression of more than 10 % compared with preoperative levels. The mean OA score was 1.57 (median 1.5) for TPLO and 1.55 (median 1.55) for TTA.

**Figure 1:**
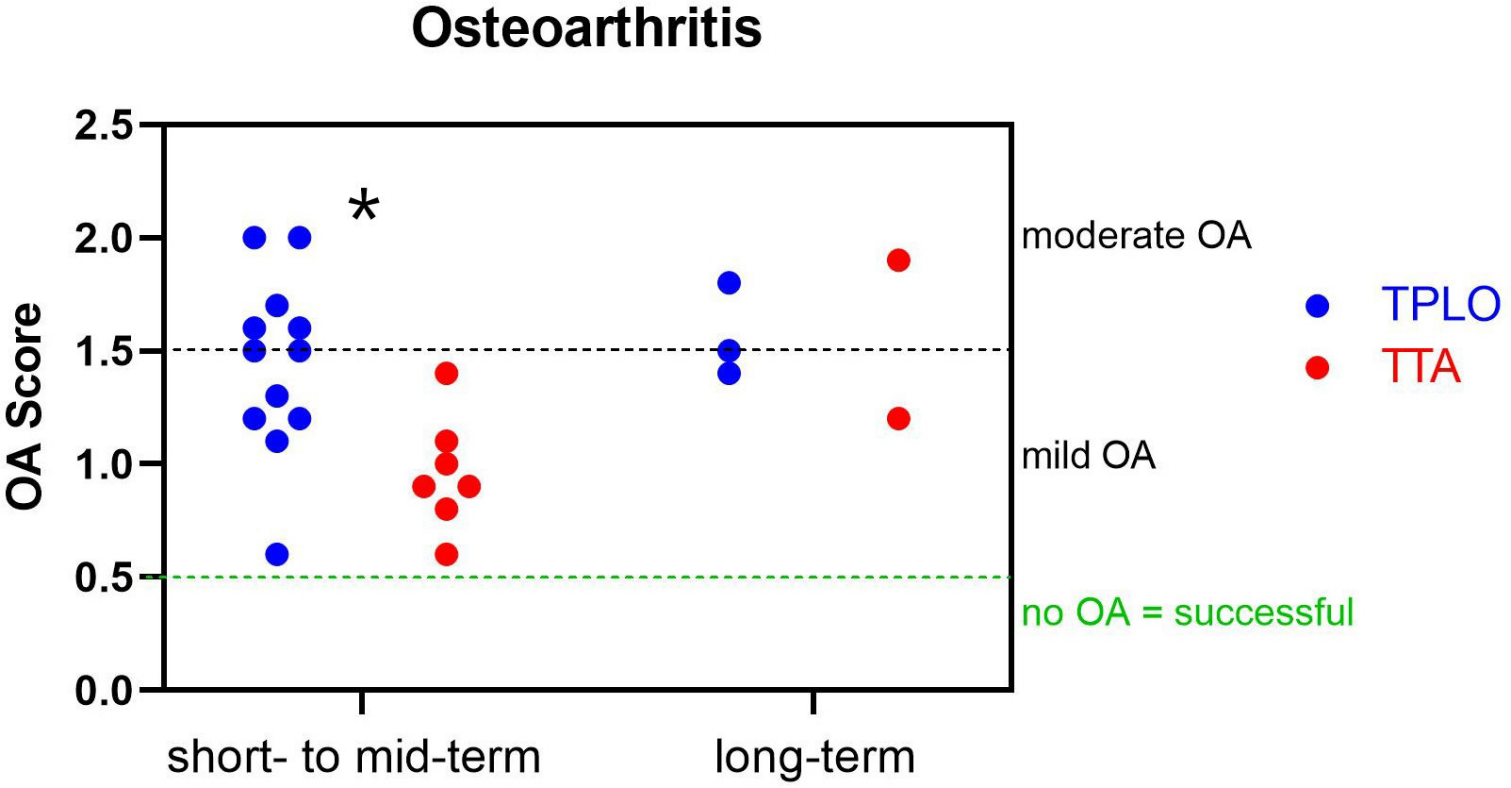
Outcome on osteoarthritis. Mean osteoarthritis scores in short- to mid-term and long-term outcome for TPLO and TTA. No study showed successful outcome in terms of no detectable osteoarthritis. The majority of the studies for TPLO and all TTA studies showed mild osteoarthritis up to 6 months postoperatively. TTA had a significant lower rate of cases developing an osteoarthritis (*P < 0.05, Mann-Whitney U-Test).

Overall, the studies showed evidence that osteoarthritis was developed radiographically after TPLO and TTA surgery at short-, mid- as well as long-term evaluation. There was a statistically significant difference in OA scores between TPLO and TTA (*p* < 0.05). After TTA, OA scores were significantly lower than after TPLO (Figure 1).

#### Thigh circumference measurements

Eight studies perform measurements of thigh circumference, five for TPLO and three for TTA surgery. In total, 316 (201 TPLO, 115 TTA) have been taken into consideration. High quality of evidence is provided in four studies on TPLO, but in no study on TTA.

Most studies reported a successful outcome, either considering that there is no significant difference in thigh circumference between the healthy and the affected leg (31, 62, 99) or that there was no loss in muscle mass after surgery (46, 66).

One study that investigated the effect of Fortetropin reported a reduction in thigh circumference between pre-operative measurement and the 8-week evaluation in the control group whereas the treatment group did not show a significant difference in muscle mass (40). Another study showed inconsistent results between treatment groups: all three groups showed statistically significant values when comparing pre-operative and post-operative levels; even though the two treatment groups showed a decrease in thigh circumference, the control group showed the opposite (39).

Overall, there was evidence that CCL disease does not have clinically relevant effects on thigh circumference in affected hind limbs after TPLO or TTA surgery.

#### Goniometry

Seven studies with a total of 189 CCL surgeries (74 TPLO, 115 TTA) reported pre- and post-operative investigations on the Range of Motion (flexion + extension) of affected joints. High quality of evidence was provided in three studies describing TPLO only.

Evidence was provided that TPLO and TTA surgery both had a successful outcome in joint mobility as the ROM, either increased (39, 66, 100) or did not change or decrease significantly (36, 57) compared to pre-operative levels, by the time of the final evaluation. However, comparison between affected and the contralateral healthy limbs showed significant differences (62, 99) with a decreased ROM in affected joints. For this outcome measure was no study with high quality of evidence for TTA, the results of TTA's effectiveness cannot be adequately determined.

#### Joint stability

Joint stability, evaluated by fluoroscopy (33, 34, 57, 93) or tibial compression test (58, 69), was assessed in six studies (four TPLO studies, one TTA study, one study comparing TPLO and TTA) with a total of 82 CCL surgeries (57 TPLO, 25 TTA). None of these studies was considered of high quality of evidence.

Fluoroscopic imaging showed that patellofemoral as well as femorotibial translation and rotation remained abnormal after TPLO surgery (33, 34, 57). While it was found that the changes in patellofemoral kinematics may be caused by an increased flexion of the stifle (33), another study did not support the findings that flexion is different from the unaffected control limb (57). However, authors agreed that there were profound changes in gait cycle in CCL affected joints even after stabilizing surgery (33, 34, 57). Cranio-caudal translation in femorotibial kinematics was also found in the fluoroscopic investigation on joints after TTA surgery (93).

In an experimental setting, one study artificially caused CCL rupture in both joints and surgically stabilized both limbs with TTA and TPLO, respectively (69). Twelve weeks post-surgery, joint stability was tested with the tibial compression test and identified a positive test (instability) in 33 % of the TTA-operated and in 13 % of the TPLO-operated stifles. When evaluating the outcome only of partial CCL rupture and therefore stable joints pre-operatively, one study finds that these joints remain stable after TPLO surgery (58).

#### Pain

Pain was assessed in six TPLO and five TTA studies with a total of 382 surgeries (169 TPLO, 213 TTA). Several validated instruments and their modifications were used to classify pain on a scale (36–39, 43, 60, 65, 66) while other authors created their own scale in expressing pain in grades (31, 100) or as absent or present (99). High quality of evidence was provided in six studies on TPLO and one of the studies on TTA.

Immediate post-operative evaluation can only be detected on TPLO studies and therefore, a comparison to TTA results was not possible. The outcome was inconsistent: in one study (38), both study groups showed pain at 24 h post-surgery whereas the other study showed successful outcome (66). All studies showed successful results regarding short-term outcome after TPLO as well as TTA (36, 38, 43, 66, 100).

There were no TPLO studies evaluating mid-term outcome of TPLO. Mid-term outcome of TTA showed that pain was still present in two of five (40 %) study groups (43, 60), while outcome was considered successful in three (60 %) groups (99, 100).

#### Complications

Complications were recorded in the majority of the studies (58 studies in total, 36 TPLO, 17 TTA and five studies comparing TPLO and TTA). Not all of these studies have been considered into the systematic analysis due to a lack of complete data (43, 79) or recorded data which only included certain types of complications (52, 56, 76, 83, 84, 86, 88, 89, 94). Some studies could not be matched to Group S, M or L as they did not provide information on mean body weight (35, 97).

The most commonly reported minor complications were surgical site infection, fibula fracture, seroma, tibial tuberosity fracture/fissure, incision dehiscence, screw loosening, plate cracks, patellar tendonitis, tissue swelling, osteomyelitis and septic arthritis (order by frequency of mention in considered studies) following TPLO and tibial tuberosity fracture/fissure, surgical site infection, incision dehiscence, implant loosening, implant rupture, seroma, tissue sweeling, patellar ligament desmitis, septic arthritis and lick granuloma following TTA. Most common major complications in both techniques were meniscal lesions, surgical site infections, tibial tuberosity fractures and implant loosing.

Most of the operated stifles were related to medium sized dogs in both TPLO and TTA. Comparing TPLO and TTA in medium sized dogs, complication rates did not differ between surgical techniques, with a mean of 20.0 % (weighted mean 22.3 %, range 0–53.3 %) in TPLO and a mean of 26.5 % (weighted mean 22.0 %, range 0–67.7 %) in TTA (Tables 5, 6).

**Table 5:** Complication rates in percent for TPLO.

**Outcome TPLO (Complication rates in %)**			
**Group S**	Minor	Major	Overall
Range	0–22.7	0	0–22.7
Mean	9.4	0	9.4
Weighted mean	10.4	0	10.4
Total no. of TPLO surgeries in group S: 212
**Group M**	Minor	Major	Overall
Range	0–53.3	0–15.4	0–53.3
Mean	18.2	3.5	20.0
Weighted mean	18.2	5.1	22.3
Total no. of TPLO surgeries in group M: 1,826
**Group L**	Minor	Major	Overall
Range	34.2–38.9	5.5–14.8	18.4–49.0
Mean	36.6	10.2	35.7
Weighted mean	36.1	11.1	25.4
Total no. of TPLO surgeries in group L: 509
**All Groups**	Minor	Major	Overall
Range	0–53.3	0–15.4	0–53.3
Mean	17.9	3.3	20.4
Weighted mean	18.1	4.8	21.9
Total no. of TPLO surgeries in all groups: 2,547

Groups S, M, and L by dogs' body weight. Complications were differentiated into minor, major, and overall complications.

**Table 6:** Complication rates in percent for TTA.

**Outcome TTA (Complication rates in %)**			
**Group S**	Minor	Major	Overall
Range	0	0–5.7	0–14.6
Mean	0	2.9	6.7
Weighted mean	0	4	9.2
Total no. of TTA surgeries in group S: 98
**Group M**	**Minor**	**Major**	**Overall**
Range	0–66.2	0–23.1	0–67.7
Mean	22.7	5.2	26.5
Weighted mean	17.1	6	22
Total no. of TTA surgeries in group M: 1,018
**Group L**	**Minor**	**Major**	**Overall**
Range	5.7–24.2	2.9–9.9	8.6–34.1
Mean	15	6.4	21.4
Weighted mean	19.1	8	27
Total no. of TTA surgeries in group L: 126
**All Groups**	**Minor**	**Major**	**Overall**
Range	0–66.2	0–23.1	0–67.7
Mean	18	5	21.6
Weighted mean	16.4	6.2	21.1
Total no. of TTA surgeries in all groups: 1,242

Groups S, M, and L by dogs' body weight. Complications were differentiated into minor, major, and overall complications.

In small dogs, TPLO surgery showed a mean complication rate of 9.4 % (weighted mean 10.4 %, range 0–22.7 %), while TTA showed a mean complication rate of 6.7 % (weighted mean 9.2 %, range 0–14.6 %) (Tables 5, 6). Due to the comparably low number and a medium to low quality of evidence, the outcome was not supported by strong evidence.

Large dogs overall had a mean complication rate of 35.7 % (weighted mean 25.4 %, range 18.4–49 %) when operated with TPLO and 21.4 % (weighted mean 27 %, range 8.6–34.1 %) when treated with TTA (Tables 5, 6). There seemed to be a higher incidence in overall complications as well as major and minor complications following TPLO surgery compared to TTA. Both TPLO and TTA studies provided a low quality of evidence, thus, any definite conclusions are precluded.

Overall, it was shown that the complication rates were comparably lower in small sized dogs. This could be observed in both TPLO and TTA surgery with no difference between the treatment option. The higher the body weight, the higher the complications rates were.

In all groups, a total of 2,547 TPLO and 1,242 TTA surgeries have been performed. The overall complication rate for TPLO was 20.4 % (weighted mean 21.9 %, range 0–53.3 %) which did not differ to the complication rate for TTA, 21.6 % (weighted mean 21.1 %, range 0–67.7 %).

#### Statistical analysis (meta-analytic approach)

##### Total complications

**Figure 2:**
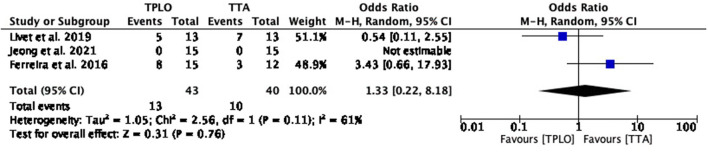
Forest plot comparing TPLO vs. TTA. Odd ratios (95% CI) of total complications for TPLO and TTA.

The common estimated OR was 1.33 (95% CI: 0.22–8.18), showing a statistically non-significant association (*P* = 0.76) between the two surgical techniques. Moderate heterogeneity was shown between studies (chi^2^ = 2.56, *P* = 0.11) (Figure 2).

##### Surgical site infection

**Figure 3:**
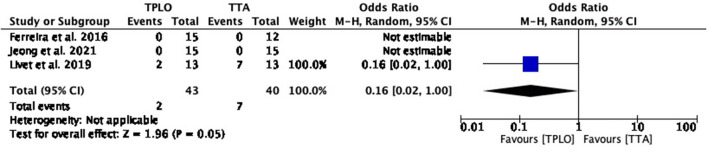
Forest plot comparing TPLO vs. TTA. Odd ratios (95% CI) of surgical site infection as a complication for TPLO and TTA.

The common estimated OR was 1.16 (95% CI: 0.02–1.00), showing a statistically significant association (*P* = 0.05) between the two surgical techniques, with reduced odds of this adverse effect in TPLO. Heterogeneity calculation was not applicable for this comparison (Figure 3).

##### Seroma formation

**Figure 4:**
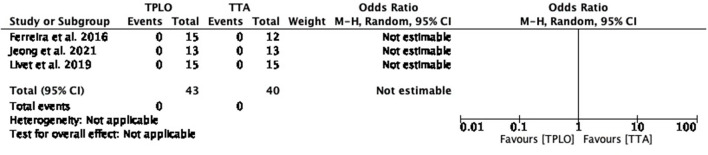
Forest plot comparing TPLO vs. TTA. Odd ratios (95% CI) of seroma formation as a complication for TPLO and TTA.

The common estimated OR could not be estimated for this complication since this complication was not reported in any of the studies (Figure 4).

##### Implant failure

**Figure 5:**
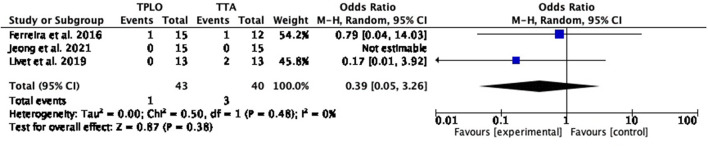
Forest plot comparing TPLO vs. TTA. Odd ratios (95% CI) of implant failure as a complication for TPLO and TTA.

The common estimated OR was 0.39 (95% CI: 0.05–3.26), and there was no association (*P* = 0.38) between the two surgical techniques. Low heterogeneity was shown between studies (chi^2^ = 0.50, *P* = 0.48) (Figure 5).

##### Late meniscal injury

**Figure 6:**
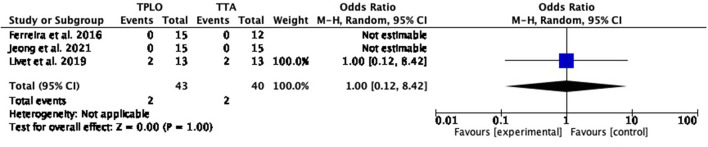
Forest plot comparing TPLO vs. TTA. Odd ratios (95% CI) of late meniscal injury as a complication for TPLO and TTA.

The common estimated OR was 1.00 (95% CI: 0.12–8.42), without a difference between the two surgical techniques (*P* = 1.00). Heterogeneity calculation was not applicable (Figure 6).

##### Tibial tuberosity fracture

**Figure 7:**
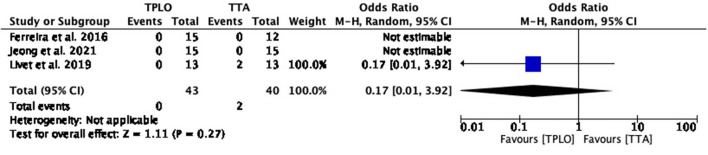
Forest plot comparing TPLO vs. TTA. Odd ratios (95% CI) of tibial tuberosity fracture as a complication for TPLO and TTA.

The common estimated OR was 0.17 (95% CI: 0.01–3.92), showing a statistically non-significant association (*P* = 0.27) between the two surgical techniques. Heterogeneity calculation was not applicable (Figure 7).

##### Fibular fracture

**Figure 8:**
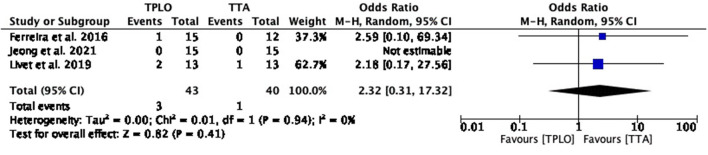
Forest plot comparing TPLO vs. TTA. Odd ratios (95% CI) of fibular fracture as a complication for TPLO and TTA.

The common estimated OR was 2.32 (95% CI: 0.31–17.32) not showing a difference between the two surgical studies (*P* = 0.41). Low heterogeneity was shown between studies (chi^2^ = 0.01, *P* = 0.94) (Figure 8).

## Discussion

This systematic review evaluated 72 studies regarding the outcome of TPLO and/or TTA, two highly relevant and common surgical interventions for CCL rupture. The results of the systematic review showed that both techniques are effective with no difference in outcome. Some former studies with either client-assessed subjective approaches (61, 101) or based on objective parameters (102) suggest that TPLO could be superior to TTA. Studies with subjective lameness assessment by a veterinarian, however, did not show differences between their outcome (48, 69), confirmed by the current study. There were also studies included that could not be evaluated within the systematic analysis as they lacked detailed data on lameness scores or scores were expressed in combination with other criteria (31, 39, 43, 49, 62, 65, 96, 97). Still, these studies confirm the assumption that both procedures can result in decreased lameness scores over time.

Using objective gait analysis measurements, studies showed that TPLO and TTA both lead to a return to normal limb function (45) with a relatively fast recovery in TTA (48) whereas one study indicated TPLO's superiority over TTA in long-term assessment (102). Our results showed significant differences in ground reaction forces at mid-term evaluation in all study groups in both TPLO and TTA indicating that both treatment options may lead to a successful outcome. However, even though a significant improvement was seen in all study groups, the improvement in ground reaction forces was often below 30 %. This might indicate that the improvement may be too small to be clinically relevant. The studies on objective gait analysis that were included use a variety of different parameters, expressed either as kinetic data, relative weight-bearing or as symmetry index. A lack of comparability can be considered when interpreting the results in the systematic approach. However, the results consistently indicated that both procedures can improve limb function when assessed with objective parameters.

Our investigations on the development of osteoarthritis confirmed recent findings (13, 23) that stifle surgery does not prevent osteoarthritis after CCL disease. However, we did not observe superiority of one technique over the other in contrast to another systematic review which reported TPLO to have a favorable effect on osteoarthritis development compared to TTA (13). Actually, our results even indicated a lower mean OA score at short- to mid-term outcome after TTA surgery and therefore a less rapid development of OA. Current investigations lack long-term results as most studies focus on a horizon up to 6 months postoperatively. Osteoarthritis usually has a long-term effect and worsens over time (23, 24). It is also affected by other factors, such as meniscal lesions (23), stability due to partial or complete rupture (24) as well as the timing of surgery as a lower stage of osteoarthritis prior to surgery is suspected to slow down osteoarthritis progression (91). Several of the included studies investigated the presence and extent of meniscal lesions during surgery (41–43), but the results do not allow conclusions whether one surgical technique is superior to the other in regard of osteoarthritis development when concurrent meniscal damage was present.

The functional outcome of joint surgery could also be assessed by goniometry and thigh circumference measurements. The affected limb may show a reduced ROM as well as a loss in muscle mass (18, 20, 103). Our findings showed improvement or at least no worsening in both parameters, however, the ROM was abnormal compared to contralateral unaffected pelvic limbs. This is also consistent with previous findings (103). Our results did not favor one surgical technique over the other regarding ROM or thigh circumference. There may be positive effects of additional treatments, which is indicated by some of the included studies (31, 36, 39, 40), but not relevant to our results.

Kinematic fluoroscopy revealed abnormal gait and instability in the stifle joint due to CCL disease (20). TPLO as well as TTA aim to alter biomechanical dynamics and restore stability, which in fact seems to be only partly achieved (20, 93). Our results showed that femorotibial, craniocaudal subluxation is still apparent after TPLO as well as TTA (57, 69, 93) with a suspected slightly higher incidence after TTA. Further research on the impact of joints' instability on the functional outcome and the underlying biomechanical alterations is needed.

We were not able to compare TPLO and TTA outcome regarding immediate post-operative or mid- to long-term pain due to a lack of availability of relevant studies and lack of or inhomogeneous data to allow a thorough statistical analysis. Short-term outcomes revealed successful results for both surgical techniques. Included studies used different approaches in assessing pain, either using established and standardized instruments (36–39, 43, 60, 65, 66) or after clinical examination stating pain is present or absent (99). There was no homogenous definition of pain in the included studies.

Previous literature indicated lower complications rates for TPLO compared to TTA (13), which is not supported by our findings. We calculated an overall mean complications rate of 20.4 % for TPLO and 21.6 % for TTA. However, if one takes a closer look at the type of complication as well as the size of the dog, differences nevertheless may become apparent. Both techniques showed higher complication rates in medium and large breed dogs, which confirmed recent findings that weight is associated with a higher risk for complications (27, 28, 71). Due to a lack of prospective, controlled, randomized and blinded studies, we were not able to perform a complete meta-analysis on outcome parameters. Three of the studies comparing TPLO and TTA however delivered sufficient data for a meta-analytic approach on complications. For the total complication rate, our meta-analytic approach confirmed that none of the surgical techniques is superior to the other. Surgical site infections were commonly reported after TPLO and TTA (59, 68, 72, 77, 82, 87, 98). Similar to previous systematic review (13) our study also favored TPLO regarding complication rates. Further research in a prospective, controlled trial is needed to verify these findings and evaluate the causes and preventive strategies. The retrospective nature of the majority of studies assessing complications carry the risk of over- or underreported complications. Owners tend to come back to the primary care hospital when a complication occurs so dogs with complications may be overreported. Dogs that do not suffer complications may not be presented again as owners do not see the need to. On the other hand, owners may be unsatisfied with the surgical outcome when facing a complication and may choose another veterinarian; thus, the primary surgeon is not informed about the complication. Another challenge is the inconsistent definition of major and minor complications. In our study, we used a standardized scale to address this inhomogeneity. However, since some authors reported the same rates for major and overall complications, it is questionable whether minor complications have been monitored and stated.

There are several limitations in our study which may preclude definite conclusions. First, the number of studies with high quality of evidence was very low. Only five of 72 included studies fulfilled all requirements to be rated as low risk of bias and there was a total of 18 studies providing high quality of evidence. Overall, due to the lack of studies with high quality of evidence, we compared TPLO and TTA outcomes using an heterogenous population of high and low quality studies. There was only one randomized, clinical trial comparing the surgical techniques of TPLO and TTA directly with each other. Retrospective design, lack of detailed outcome description as well as low numbers of included patients lead to inconsistent results and questionable comparability. Therefore, solid conclusions are precluded. Further research, especially with randomized, surgical clinical trials is required to compare surgical techniques for CCL rupture in a controlled and standardized setting.

## Conclusion

TPLO and TTA are both effective surgical approaches to treat naturally occurring CCL disease/rupture. There is evidence that both techniques provide good outcome and restore functionality and mobility for affected canines. However, long-term development of osteoarthritis must be considered and long-term follow up data are spare. There is no clear recommendation regarding the choice of one technique over the other, but the current evidence indicates that TPLO is favorable regarding certain complications, such as SSIs. Further research providing high quality of evidence is vital to confirm our findings and further assess the differences in outcome parameters between the two surgical interventions.

## Data availability statement

The original contributions presented in the study are included in the article/Supplementary material, further inquiries can be directed to the corresponding author.

## Author contributions

AW collected the data for the study, assessed the data, and wrote the first draft of the manuscript. MC and HV performed statistical analysis and meta-analytic approach. All authors helped to draft the manuscript, participated in its design, read, and approved the final manuscript.

## Funding

This Open Access publication was funded by the Deutsche Forschungsgemeinschaft (DFG, German Research Foundation) 491094227 Open Access Publication Costs and the University of Veterinary Medicine Hannover, Foundation.

## Conflict of interest

The authors declare that the research was conducted in the absence of any commercial or financial relationships that could be construed as a potential conflict of interest.

## Publisher's note

All claims expressed in this article are solely those of the authors and do not necessarily represent those of their affiliated organizations, or those of the publisher, the editors and the reviewers. Any product that may be evaluated in this article, or claim that may be made by its manufacturer, is not guaranteed or endorsed by the publisher.

## Abbreviations

AB: Attrition bias
bRCT(s): Blinded randomized clinical trial(s)
CCL: Cranial cruciate ligament
CCS: Case-control study(ies)
CI: Confidence Interval
CoS: Cohort study(ies)
CR: Case report(s)
CS: Case series
DB: Detection bias
L: Large
M: Medium
nbRCT(s): Non-blinded randomized clinical trial(s)
No.: Number
NRCT(s): Non-randomized clinical trial(s)
OA: Osteoarthritis
ObS: Observational studies
OR: Odds Ratio
PB: Performance bias
RB: Reporting bias
ROM: Range of Motion
S: Small
SB A: Selection bias (allocation concealment)
SB R: Selection bias (random sequence generation
SSI: Surgical site infection
TPLO: Tibial Plateau Leveling Osteotomy
TTA: Tibial Tuberosity Advancement
UCT(s): Uncontrolled clinical trial(s).
