# Supplementary material for: Surgical treatment of cranial cruciate ligament disease in dogs using Tibial Plateau Leveling Osteotomy or Tibial Tuberosity Advancement–A systematic review with a meta-analytic approach
Source: Front Vet Sci. 2022 Nov 30;9:1004637. doi: 10.3389/fvets.2022.1004637 (PMC9748159; doi:10.3389/fvets.2022.1004637)
Supplement: Supplementary file 1 [file Table_1.pdf]

Table 1: Quality assessment of the included studies regarding study type, group size, duration of the study, study enrolment quality and overall risk of bias. Each risk of bias is rated on a scale between 3 = high risk of bias, 2 = moderate or unclear risk of bias and 1 = low risk of bias
**SB R" = Random sequence generation (selection bias); "SB A" = Allocation concealment (selection bias); "PB" = Blinding of participants and personnel (performance bias); "DB" = Blinding of outcome assessment (detection bias); "AB" = Incomplete outcome data addressed (attrition bias); "RB" = Selective reporting (reporting bias*

Ref.	Year	Author	Group	Study type	Group size	Duration of the study	Study enrolment quality	SB R*	SB A*	PB*	DB*	AB*	RB*	Other	Total	Overall risk of bias
1	2018	Spencer DD, Raye RM	A	bRCT	good	short-term	well	1	1	1	1	1	1	1	7	low
2	2018	Kennedy et al.	A	bRCT	very small	short-term	well	1	1	1	1	1	1	2	8	low
3	2017	Rogatko et al.	A	bRCT	small	short-term	well	1	1	1	1	2	1	2	9	low
4	2017	von Freeden et al.	A	bRCT	small	short-term	well	1	1	1	1	1	2	2	9	low
5	2020	White et al.	A	bRCT	moderate	mid-term	unclear	1	1	1	1	2	1	2	9	low
6	2018	Heffernan et al.	A	bRCT	small	short-term	unclear	1	1	2	1	1	2	2	10	low/moderate
7	2018	Kieves et al.	A	bRCT	moderate	mid-term	well	2	2	1	1	1	1	2	10	low/moderate
8	2018	Renwick et al.	A	bRCT	moderate	short-term	well	1	1	1	1	1	3	2	10	low/moderate
9	2021	Serra Aguado et al.	A	bRCT	moderate	mid-term	unclear	1	1	2	1	1	2	2	10	low/moderate
10	2018	Baltzer et al.	A	bRCT	small	mid-term	well	1	1	2	2	2	1	2	11	low/moderate
11	2018	Verpaalen et al.	A	bRCT	small	mid-term	well	2	2	2	1	2	1	2	12	low/moderate
12	2019	Barnes et al.	A	nbRCT	very small	short-term	well	1	1	3	3	2	1	1	12	low/moderate
13	2019	Livet et al.	A	nbRCT	small	mid-term	well	1	1	3	2	2	2	1	12	low/moderate
14	2021	Macri et al.	A	nbRCT	small	long-term	well	2	2	3	1	2	2	1	13	moderate
15	2017	Martini et al.	A	nbRCT	very small	mid-term	well	2	2	3	3	1	1	2	14	moderate
16	2016	Barnhart et al.	A	nbRCT	moderate	mid-term	fairly	1	1	3	3	2	2	2	14	moderate
17	2020	Knebel et al.	A	nbRCT	moderate	mid-term	well	2	3	3	3	1	2	2	16	moderate
18	2019	Giannetto JJ, Aktay SA	A	nbRCT	good	mid-term	unclear	2	3	3	2	2	3	1	16	moderate
19	2021	Jeong et al.	B	NRCT	small	mid-term	well	2	1	3	2	1	1	2	12	moderate

20	2016	Ferreira et al.	B	NRCT	small	mid-term	well	3	3	3	3	1	1	1	15	moderate
21	2020	Bernardi et al.	B	UCT	good	mid-term	fairly	3	3	3	3	1	1	1	15	moderate
22	2019	Ferreira et al.	B	UCT	moderate	mid-term	well	3	3	3	3	1	1	1	15	moderate
23	2020	Fujino et al.	B	UCT	moderate	short-term	well	3	3	3	3	1	1	1	15	moderate
24	2019	Amimoto H, Koreeda T	B	UCT	small	long-term	well	3	3	3	3	1	1	1	15	moderate
25	2018	Gomes et al.	B	UCT	moderate	mid-term	well	3	3	3	3	1	2	1	16	moderate/high
26	2017	Arican et al.	B	UCT	small	mid-term	fairly	3	3	3	2	1	2	2	16	moderate/high
27	2019	Frank et al.	B	UCT	very small	short-term	unclear	3	3	3	3	1	1	2	16	moderate/high
28	2017	Kim et al.	B	UCT	small	mid-term	well	3	3	3	3	1	2	2	17	moderate/high
29	2016	Aydin Kaya D, Altunatmaz K	B	UCT	small	mid-term	fairly	3	3	3	3	1	2	2	17	moderate/high
30	2020	Tinga et al.	B	UCT	small	mid-term	well	3	3	3	3	2	1	2	17	moderate/high
31	2016	Barger et al.	B	UCT	very small	long-term	well	3	3	3	3	2	1	3	18	moderate/high
32	2016	Medeiros et al.	B	UCT	moderate	mid-term	fairly	3	3	3	3	2	2	2	18	moderate/high
33	2020	Amimoto et al.	B	UCT	small	mid-term	well	3	3	3	3	2	1	3	18	moderate/high
34	2020	Zann et al.	B	UCT	small	mid-term	poorly	3	3	3	3	2	2	2	18	moderate/high
35	2017	Butterworth SJ, Kydd DM	B	UCT	good	long-term	unclear	3	3	3	3	2	2	3	19	high
36	2017	Dyall B, Schmökel H	B	UCT	moderate	short-term	well	3	3	3	3	2	3	2	19	high
37	2017	Bureau S	B	UCT	good	long-term	fairly	3	3	3	3	2	3	3	20	high
38	2018	Medeiros et al.	B	UCT	small	long-term	unclear	3	3	3	2	3	3	3	20	high
39	2018	Löfqvist et al.	C	ObS	small	short-term	well	3	3	3	2	2	2	2	17	moderate/high
40	2021	Polajnar et al.	C	CS	moderate	short-term	well	3	3	3	2	2	2	3	18	moderate/high
41	2021	Geier et al.	C	CoS	moderate	mid-term	unclear	3	3	3	2	2	2	3	18	moderate/high
42	2019	Ferrell et al.	C	CS	good	long-term	unclear	3	3	3	3	2	2	3	19	high
43	2018	Morgan MJ, Frazho JK	C	CoS	good	short-term	unclear	3	3	3	3	2	2	3	19	high

44	2019	Pinna et al.	C	CS	moderate	mid-term	fairly	3	3	3	3	2	2	3	19	high
45	2018	Retallack LM, Daye RM	C	CoS	moderate	short-term	well	3	3	3	3	2	2	3	19	high
46	2018	Schwede et al.	C	CS	very small	short-term	well	3	3	3	3	2	2	3	19	high
47	2018	Selmic et al.	C	CCS	moderate	long-term	unclear	2	2	3	3	3	3	3	19	high
48	2018	Stine et al.	C	CoS	good	long-term	unclear	3	3	3	3	2	2	3	19	high
49	2016	Barnes et al.	C	CS	moderate	short-term	unclear	3	3	3	3	2	2	3	19	high
50	2017	Costa et al.	C	CS	good	long-term	unclear	3	3	3	3	2	2	3	19	high
51	2019	McGregor et al.	C	CS	good	mid-term	unclear	3	3	3	2	3	2	3	19	high
52	2018	Mehrkens et al.	C	CCS	moderate	short-term	unclear	3	3	3	3	2	2	3	19	high
53	2019	Tuan et al.	C	CoS	moderate	long-term	unclear	3	3	3	3	2	3	3	20	high
54	2016	Brown et al.	C	CS	good	long-term	unclear	3	3	3	3	2	3	3	20	high
55	2019	Chiu et al.	C	CS	moderate	short-term	well	3	3	3	3	3	2	3	20	high
56	2016	Dantas et al.	C	CS	moderate	mid-term	well	3	3	3	3	3	2	3	20	high
57	2021	Engdahl et al.	C	CoS	good	short-term	unclear	3	3	3	3	2	3	3	20	high
58	2020	Hagen et al.	C	CS	good	short-term	unclear	3	3	3	3	2	3	3	20	high
59	2017	Hans et al.	C	CoS	moderate	long-term	well	3	3	3	3	2	3	3	20	high
60	2021	Peress et al.	C	CoS	good	short-term	well	3	3	3	3	3	2	3	20	high
61	2017	Seo et al.	C	CR	very small	long-term	well	3	3	3	3	2	3	3	20	high
62	2018	Wilson et al.	C	CS	good	short-term	unclear	3	3	3	3	3	2	3	20	high
63	2019	Trisciuzzi et al.	C	CS	moderate	mid-term	fairly	3	3	3	3	2	3	3	20	high
64	2019	Cappelle KK, Barnhart MD	C	CS	small	short-term	unclear	3	3	3	3	3	2	3	20	high
65	2020	Clark et al.	C	CS	moderate	long-term	unclear	3	3	3	3	3	2	3	20	high
66	2019	Crovace et al.	C	CS	very small	mid-term	unclear	3	3	3	3	3	2	3	20	high
67	2018	Knight R, Danielski A	C	CS	good	long-term	well	3	3	3	3	2	3	3	20	high

68	2018	Lopez et al.	C	CoS	good	short-term	unclear	3	3	3	3	3	2	3	20	high
69	2021	Marin et al.	C	CS	good	short-term	well	3	3	3	3	3	2	3	20	high
70	2021	McDougall et al.	C	CCS	good	short-term	unclear	3	3	3	3	3	2	3	20	high
71	2020	Moore et al.	C	CoS	moderate	long-term	unclear	3	3	3	3	3	3	3	21	high
72	2021	Matchwick et al	C	CS	good	long-term	well	3	3	3	3	3	3	3	21	high

References

1. Spencer DD, Daye RM. A prospective, randomized, double-blinded, placebo-controlled clinical study on postoperative antibiotherapy in 150 arthroscopy-assisted tibial plateau leveling osteotomies in dogs. *Vet Surg*. Nov 2018;47(8):E79-E87. doi:10.1111/vsu.12958
2. Kennedy KC, Martinez SA, Martinez SE, Tucker RL, Davies NM. Effects of low-level laser therapy on bone healing and signs of pain in dogs following tibial plateau leveling osteotomy. United States: AVMA AMERICAN VETERINARY MEDICAL ASSOCIATION; 2018. p. 893.
3. Rogatko CP, Baltzer WI, Tennant R. Preoperative low level laser therapy in dogs undergoing tibial plateau levelling osteotomy: A blinded, prospective, randomized clinical trial. *Vet Comp Orthop Traumatol*. Jan 16 2017;30(1):46-53. doi:10.3415/VCOT-15-12-0198
4. von Freeden N, Duerr F, Fehr M, Diekmann C, Mandel C, Harms O. Comparison of two cold compression therapy protocols after tibial plateau leveling osteotomy in dogs. *Tierarztl Prax Ausg K Kleintiere Heimtiere*. Aug 10 2017;45(4):226-233. doi:10.15654/TPK-170049
5. White DA, Harkin KR, Roush JK, Renberg WC, Biller D. Fortetropin inhibits disuse muscle atrophy in dogs after tibial plateau leveling osteotomy. *PLoS One*. 2020;15(4):e0231306. doi:10.1371/journal.pone.0231306
6. Heffernan AE, Katz EM, Sun Y, Rendahl AK, Conzemius MG. Once daily oral extended-release hydrocodone as analgesia following tibial plateau leveling osteotomy in dogs. *Vet Surg*. May 2018;47(4):516-523. doi:10.1111/vsu.12792
7. Kieves NR, Canapp SO, Lotsikas PJ, et al. Effects of low-intensity pulsed ultrasound on radiographic healing of tibial plateau leveling osteotomies in dogs: a prospective, randomized, double-blinded study. *Vet Surg*. Jul 2018;47(5):614-622. doi:10.1111/vsu.12798
8. Renwick SM, Renwick AI, Brodbelt DC, Ferguson J, Abreu H. Influence of class IV laser therapy on the outcomes of tibial plateau leveling osteotomy in dogs. *Vet Surg*. May 2018;47(4):507-515. doi:10.1111/vsu.12794
9. Serra Aguado CI, Ramos-Pla JJ, Soler C, Segarra S, Moratalla V, Redondo JI. Effects of Oral Hyaluronic Acid Administration in Dogs Following Tibial Tuberosity Advancement Surgery for Cranial Cruciate Ligament Injury. *Animals (Basel)*. Apr 27 2021;11(5)doi:10.3390/ani11051264
10. Baltzer WI, Smith-Ostrin S, Warnock JJ, Ruaux CG. Evaluation of the clinical effects of diet and physical rehabilitation in dogs following tibial plateau leveling osteotomy. Journal Article. *Journal of the American Veterinary Medical Association*. // 2018;252(6):686-700. doi:10.2460/javma.252.6.686

http://www.avma.org

11. Verpaalen VD, Baltzer WI, Smith-Ostrin S, Warnock JJ, Stang B, Ruaux CG. Assessment of the effects of diet and physical rehabilitation on radiographic findings and markers of synovial inflammation in dogs following tibial plateau leveling osteotomy. Journal Article. *Journal of the American Veterinary Medical Association*. // 2018;252(6):701-709. doi:10.2460/javma.252.6.701

http://www.avma.org

12. Barnes K, Faludi A, Takawira C, et al. Extracorporeal shock wave therapy improves short-term limb use after canine tibial plateau leveling osteotomy. *Vet Surg*. Nov 2019;48(8):1382-1390. doi:10.1111/vsu.13320

13. Livet V, Baldinger A, Viguier E, et al. Comparison of Outcomes Associated with Tibial Plateau Levelling Osteotomy and a Modified Technique for Tibial Tuberosity Advancement for the Treatment of Cranial Cruciate Ligament Disease in Dogs: A Randomized Clinical Study. *Vet Comp Orthop Traumatol*. Jul 2019;32(4):314-323. doi:10.1055/s-0039-1684050
14. Macri F, Cicero L, Angileri V, et al. Locking compression plates versus locking plates for tibial plateau levelling osteotomy in dogs: progression of osteoarthritis, bone healing score and lameness degree. *BMC Vet Res*. May 13 2021;17(1):193. doi:10.1186/s12917-021-02899-6
15. Martini FM, Brandstetter de Bellesini A, Miolo A, Del Coco L, Fanizzi FP, Crovace A. Combining a joint health supplement with tibial plateau leveling osteotomy in dogs with cranial cruciate ligament rupture. An exploratory controlled trial. *Int J Vet Sci Med*. Dec 2017;5(2):105-112. doi:10.1016/j.ijvsm.2017.09.006
16. Barnhart MD, Watson AT, Thatcher LG, Wotton H, Naber SJ. Prospective Randomized Clinical and Radiographic Evaluation of a Novel Bioabsorbable Biocomposite Tibial Tuberosity Advancement Cage Implant. *Vet Surg*. Jul 2016;45(5):651-8. doi:10.1111/vsu.12502
17. Knebel J, Eberle D, Steigmeier-Raith S, Reese S, Meyer-Lindenberg A. Outcome after Tibial Plateau Levelling Osteotomy and Modified Maquet Procedure in Dogs with Cranial Cruciate Ligament Rupture. *Vet Comp Orthop Traumatol*. May 2020;33(3):189-197. doi:10.1055/s-0040-1701502
18. Giannetto JJ, Aktay SA. Prospective Evaluation of Surgical Wound Dressings and the Incidence of Surgical Site Infections in Dogs Undergoing a Tibial Plateau Levelling Osteotomy. *Vet Comp Orthop Traumatol*. Jan 2019;32(1):18-25. doi:10.1055/s-0038-1676352
19. Jeong J, Jeong SM, Kim SE, Lewis DD, Lee H. Subsequent meniscal tears following tibial tuberosity advancement and tibial plateau leveling osteotomy in dogs with cranial cruciate ligament deficiency: An in vivo experimental study. *Vet Surg*. Jul 2021;50(5):966-974. doi:10.1111/vsu.13648
20. Ferreira MP, Ferrigno CR, de Souza AN, Caquias DF, de Figueiredo AV. Short-term comparison of tibial tuberosity advancement and tibial plateau levelling osteotomy in dogs with cranial cruciate ligament disease using kinetic analysis. *Vet Comp Orthop Traumatol*. May 18 2016;29(3):209-13. doi:10.3415/VCOT-15-01-0009
21. Bernardi-Villavicencio C, Jimenez-Socorro AN, Rojo-Salvador C, Robles-Sanmartin J, Rodriguez-Quiros J. Short-term outcomes and complications of 65 cases of porous TTA with flange: a prospective clinical study in dogs. *BMC Vet Res*. Aug 10 2020;16(1):279. doi:10.1186/s12917-020-02469-2
22. Ferreira AJA, Bom RM, Tavares SO. Tibial tuberosity advancement technique in small breed dogs: study of 30 consecutive dogs (35 stifles). *J Small Anim Pract*. May 2019;60(5):305-312. doi:10.1111/jsap.12991
23. Fujino H, Honnami M, Mochizuki M. Preoperative planning for tibial plateau leveling osteotomy based on proximal tibial width. *J Vet Med Sci*. May 20 2020;82(5):661-667. doi:10.1292/jvms.19-0501
24. Amimoto H, Koreeda T. Evaluation of recovery of limb function by use of force plate gait analysis after tibial plateau leveling osteotomy for management of dogs with unilateral cranial cruciate ligament rupture. Journal Article. *American Journal of Veterinary Research*. // 2019;80(5):461-468. doi:10.2460/ajvr.80.5.461

https://avmajournals.avma.org/doi/abs/10.2460/ajvr.80.5.461
25. Gomes Junior DC, Oriá AP, Vieira JVR, Barbosa SF, Estrela-Lima A, Dórea Neto FA. Using allogeneic cortical graft preserved in glycerin as spacer in the advancement of tibial tuberosity in 34 dogs. *Pesquisa Veterinária Brasileira*. 2018;38(12):2246-2253. doi:10.1590/1678-5150-pvb-5968
26. Arican M, Parlak K, Şahin H. Evaluation and application of the TTA-rapid method in dogs with cranial cruciate ligament rupture. *Acta Veterinaria*. 2017;67(2):238-253. doi:10.1515/acve-2017-0020
27. Frank I, Duerr F, Zanghi B, Middleton R, Lang L. Diagnostic Ultrasound Detection of Changes in Femoral Muscle Mass Recovery after Tibial Plateau Levelling Osteotomy in Dogs. *Vet Comp Orthop Traumatol*. Sep 2019;32(5):394-400. doi:10.1055/s-0039-1688985
28. Kim SE, Zann GJ, Tinga S, Moore EJ, Pozzi A, Banks SA. Patellofemoral kinematics in dogs with cranial cruciate ligament insufficiency: an in-vivo fluoroscopic analysis during walking. *BMC Vet Res*. Aug 17 2017;13(1):250. doi:10.1186/s12917-017-1186-1
29. Aydin Kaya D, Altunatmaz K. The Clinical and Radiological Evaluation of Canine Cranial Cruciate Ligament Rupture Treatment with Tibial Plateau Leveling Osteotomy. *Journal of the Faculty of*

Veterinary Medicine Istanbul University. 2015;42(1)doi:10.16988/iuvfd.2016.50881
30. Tinga S, Kim SE, Banks SA, et al. Femorotibial kinematics in dogs treated with tibial plateau leveling osteotomy for cranial cruciate ligament insufficiency: An in vivo fluoroscopic analysis during walking. *Vet Surg*. Jan 2020;49(1):187-199. doi:10.1111/vsu.13356
31. Barger B, Piazza A, Muir P. Treatment of stable partial cruciate rupture (Grade 1 sprain) in five dogs with tibial plateau levelling osteotomy. *Veterinary Record Case Reports*. 2016;4(1)doi:10.1136/vetreccr-2016-000315
32. Medeiros RM, Silva MAM, Teixeira PPM, et al. Use of castor bean polymer in developing a new technique for tibial tuberosity advancement for cranial cruciate ligament rupture correction in dogs. *Veterinární Medicína*. 2016;61(No. 7):382-388. doi:10.17221/168/2015-vetmed

33. Amimoto H, Koreeda T, Ochi Y, et al. Force Plate Gait Analysis and Clinical Results after Tibial Plateau Levelling Osteotomy for Cranial Cruciate Ligament Rupture in Small Breed Dogs. *Vet Comp Orthop Traumatol*. May 2020;33(3):183-188. doi:10.1055/s-0039-1700990
34. Zann GJ, Kim SE, Tinga S, Pozzi A, Banks SA. The effect of tibial plateau leveling osteotomy on patellofemoral kinematics in dogs: An in vivo study. *Vet Surg*. Jan 2020;49(1):207-213. doi:10.1111/vsu.13300
35. Butterworth SJ, Kydd DM. TTA-Rapid in the treatment of the canine cruciate deficient stifle: short- and medium-term outcome. *J Small Anim Pract*. Jan 2017;58(1):35-41. doi:10.1111/jsap.12610
36. Dyall B, Schmokel H. Tibial tuberosity advancement in small-breed dogs using TTA Rapid implants: complications and outcome. *J Small Anim Pract*. Jun 2017;58(6):314-322. doi:10.1111/jsap.12654
37. Bureau S. Owner assessment of the outcome of tibial plateau levelling osteotomy without meniscal evaluation for treatment of naturally occurring cranial cruciate ligament rupture: 130 cases (2009 to 2013). *J Small Anim Pract*. Aug 2017;58(8):468-475. doi:10.1111/jsap.12691
38. Medeiros RM, Silva MAM, Teixeira PPM, et al. Long-term assessment of a modified tibial tuberosity advancement technique in dogs. Journal Article. *Arquivo Brasileiro de Medicina Veterinária e Zootecnia*. // 2018;70(4):1053-1059. doi:10.1590/1678-4162-9594

http://www.scielo.br/scielo.php?script=sci_arttext&pid=S0102-09352018000401053&lng=en&nrm=iso&tlng=en
39. Lofqvist K, Kjelgaard-Hansen M, Nielsen MBM. Usefulness of C-reactive protein and serum amyloid A in early detection of postoperative infectious complications to tibial plateau leveling osteotomy in dogs. *Acta Vet Scand*. May 21 2018;60(1):30. doi:10.1186/s13028-018-0385-5
40. Polajnar P, Szanto Z, Gruborovic S, Willmitzer F, Medl N. Tibial plateau levelling osteotomy using a dome-shaped saw blade for canine cranial cruciate ligament insufficiency. *Vet Rec*. May 2021;188(10):e241. doi:10.1002/vetr.241
41. Geier CM, Frederick SW, Cross AR. Evaluation of the risk of patella fracture as the result of decreasing tibial plateau angle following tibial plateau leveling osteotomy. *Vet Surg*. Jul 2021;50(5):984-989. doi:10.1111/vsu.13640
42. Ferrell CL, Barnhart MD, Herman E. Impact of postoperative antibiotics on rates of infection and implant removal after tibial tuberosity advancement in 1,768 canine stifles. *Vet Surg*. Jul 2019;48(5):694-699. doi:10.1111/vsu.13250
43. Morgan MJ, Frazho JK. Comparison of TPLO tibial tuberosity fractures with and without an in situ rotational pin. *BMC Res Notes*. Jun 8 2018;11(1):368. doi:10.1186/s13104-018-3474-7
44. Pinna S, Lanzi F, Cordella A, Diana A. Relationship between the stage of osteoarthritis before and six months after tibial tuberosity advancement procedure in dogs. *PLoS One*. 2019;14(8):e0219849. doi:10.1371/journal.pone.0219849
45. Retallack LM, Daye RM. A modified Maquet-tibial tuberosity advancement technique for treatment of canine cranial cruciate ligament disease: Short term outcome and complications. *Vet Surg*. Jan 2018;47(1):44-51. doi:10.1111/vsu.12733
46. Schwede M, Rey J, Bottcher P. In vivo fluoroscopic kinematography of cranio-caudal stifle stability after tibial tuberosity advancement (TTA): a retrospective case series of 10 stifles. *Open Vet J*. 2018;8(3):295-304. doi:10.4314/ovj.v8i3.8
47. Selmic LE, Ryan SD, Ruple A, Pass WE, Withrow SJ. Association of tibial plateau leveling osteotomy with proximal tibial osteosarcoma in dogs. Journal Article. *Journal of the American Veterinary Medical Association*. // 2018;253(6):752-756. doi:10.2460/javma.253.6.752

https://avmajournals.avma.org/loi/javma/
48. Stine SL, Odum SM, Mertens WD. Protocol changes to reduce implant-associated infection rate after tibial plateau leveling osteotomy: 703 dogs, 811 TPLO (2006-2014). *Vet Surg*. May 2018;47(4):481-489. doi:10.1111/vsu.12796
49. Barnes DC, Trinterud T, Owen MR, Bush MA. Short-term outcome and complications of TPLO using anatomically contoured locking compression plates in small/medium-breed dogs with "excessive" tibial plateau angle. *J Small Anim Pract*. Jun 2016;57(6):305-10. doi:10.1111/jsap.12486
50. Costa M, Craig D, Cambridge T, Sebestyen P, Su Y, Fahie MA. Major complications of tibial tuberosity advancement in 1613 dogs. *Vet Surg*. May 2017;46(4):494-500. doi:10.1111/vsu.12649
51. McGregor RE, Buffa EA, Tan CJ, Schembri MA, Badcock CA, Lai A. A Retrospective Study Using the String of Pearls Tibial Plateau Levelling Osteotomy Locking Plate for the Treatment of Cranial Cruciate Ligament Disease. *Vet Comp Orthop Traumatol*. Nov 2019;32(6):483-491. doi:10.1055/s-0039-1692189
52. Mehrkens LR, Hudson CC, Cole GL. Factors associated with early tibial tuberosity fracture after tibial plateau leveling osteotomy. *Vet Surg*. Jul 2018;47(5):634-639. doi:10.1111/vsu.12915

53. Tuan J, Solano MA, Danielski A. Risk of infection after double locking plate and screw fixation of tibial plateau leveling osteotomies in dogs weighing greater than 50 kilograms. *Vet Surg*. Oct 2019;48(7):1211-1217. doi:10.1111/vsu.13308
54. Brown G, Maddox T, Baglietto Siles MM. Client-assessed long-term outcome in dogs with surgical site infection following tibial plateau levelling osteotomy. *Vet Rec*. Oct 22 2016;179(16):409. doi:10.1136/vr.103688
55. Chiu KW, Amsellem PM, Yu J, Ho PS, Radasch R. Influence of fixation systems on complications after tibial plateau leveling osteotomy in dogs greater than 45.4 kilograms (100 lb). *Vet Surg*. May 2019;48(4):505-512. doi:10.1111/vsu.13151
56. de Lima Dantas B, Sul R, Parkin T, Calvo I. Incidence of complications associated with tibial tuberosity advancement in Boxer dogs. *Vet Comp Orthop Traumatol*. 2016;29(1):39-45. doi:10.3415/VCOT-15-02-0036
57. Engdahl KS, Boge GS, Bergstrom AF, Moldal ER, Hoglund OV. Risk factors for severe postoperative complications in dogs with cranial cruciate ligament disease - A survival analysis. *Prev Vet Med*. Apr 16 2021;191:105350. doi:10.1016/j.prevetmed.2021.105350
58. Hagen CRM, Singh A, Weese JS, Marshall Q, Linden AZ, Gibson TWG. Contributing factors to surgical site infection after tibial plateau leveling osteotomy: A follow-up retrospective study. *Vet Surg*. Jul 2020;49(5):930-939. doi:10.1111/vsu.13436
59. Hans EC, Barnhart MD, Kennedy SC, Naber SJ. Comparison of complications following tibial tuberosity advancement and tibial plateau levelling osteotomy in very large and giant dogs 50 kg or more in body weight. *Vet Comp Orthop Traumatol*. Jul 20 2017;30(4):299-305. doi:10.3415/VCOT-16-07-0106
60. Peress R, Mejia S, Unis M, Sotgiu G, Dore S, Bruecker K. Comparison of Intra- and Postoperative Complications between Bilateral Simultaneous and Staged Tibial Plateau Levelling Osteotomy with Arthroscopy in 176 Cases. *Vet Comp Orthop Traumatol*. Mar 2021;34(2):91-98. doi:10.1055/s-0040-1716682
61. Seo S, Rahman M, Jeong I. Importance of meniscal injury diagnosis and surgical management in dogs during reconstruction of cranial cruciate ligament rupture: A retrospective study. *Journal of Advanced Veterinary and Animal Research*. 2017;4(3)doi:10.5455/javar.2017.d223
62. Wilson ML, Roush JK, Renberg WC. Comparison of the Effect of Dog, Surgeon and Surgical Procedure Variables on Improvement in Eight-Week Static Weight-Bearing following Tibial Plateau Levelling Osteotomy. *Vet Comp Orthop Traumatol*. Nov 2018;31(6):396-404. doi:10.1055/s-0038-1667139
63. Trisciuzzi R, Fracassi L, Martin HA, et al. 41 Cases of Treatment of Cranial Cruciate Ligament Rupture with Porous TTA: Three Years of Follow Up. *Vet Sci*. Feb 20 2019;6(1)doi:10.3390/vetsci6010018
64. Cappelle KK, Barnhart MD. Short-Term Complications following Single-Session versus Staged Bilateral Tibial Plateau Levelling Osteotomies Stabilized with Locking Plates for Treatment of Bilateral Cranial Cruciate Ligament Disease: A Retrospective Study. *Vet Comp Orthop Traumatol*. Nov 2019;32(6):460-466. doi:10.1055/s-0039-1693153
65. Clark AC, Greco JJ, Bergman PJ. Influence of administration of antimicrobial medications after tibial plateau leveling osteotomy on surgical site infections: A retrospective study of 308 dogs. *Vet Surg*. Jan 2020;49(1):106-113. doi:10.1111/vsu.13337
66. Crovace AM, Staffieri F, Monopoli D, et al. Role of Tibial Tuberosity Fracture/Fissure through the Maquet Hole in Stifle Osteoarthritis after Porous Tibial Tuberosity Advancement in Dogs at Mid-Term Follow-Up. *Vet Sci*. Dec 22 2019;7(1)doi:10.3390/vetsci7010001
67. Knight R, Danielski A. Long-term complications following tibial plateau levelling osteotomy in small dogs with tibial plateau angles > 30 degrees. *Vet Rec*. Apr 21 2018;182(16):461. doi:10.1136/vr.104491
68. Lopez DJ, VanDeventer GM, Krotscheck U, et al. Retrospective study of factors associated with surgical site infection in dogs following tibial plateau leveling osteotomy. United States: AVMA AMERICAN VETERINARY MEDICAL ASSOCIATION; 2018. p. 315.
69. Marin K, Unis MD, Horgan JE, Roush JK. Risk factors for short-term postoperative complications in the 8 weeks after tibial plateau leveling osteotomy in dogs weighing less than 15 kilograms: A retrospective study. *PLoS One*. 2021;16(2):e0247555. doi:10.1371/journal.pone.0247555
70. McDougall RA, Spector DI, Hart RC, Dycus DL, Erb HN. Timing of and risk factors for deep surgical site infection requiring implant removal following canine tibial plateau leveling osteotomy. *Vet Surg*. Jul 2021;50(5):999-1008. doi:10.1111/vsu.13634
71. Moore EV, Weeren R, Paek M. Extended long-term radiographic and functional comparison of tibial plateau leveling osteotomy vs tibial tuberosity advancement for cranial cruciate ligament rupture in the dog. *Vet Surg*. Jan 2020;49(1):146-154. doi:10.1111/vsu.13277
72. Matchwick AIM, Bridges JP, Scrimgeour AB, Worth AJ. A retrospective evaluation of complications associated with forkless tibial tuberosity advancement performed in primary care practice. *Vet Surg*. Jan 2021;50(1):121-132. doi:10.1111/vsu.13502
